# Effects of smartphone use while walking on external knee abduction moment peak: A crossover randomized trial on an instrumented treadmill

**DOI:** 10.1016/j.heliyon.2023.e21163

**Published:** 2023-10-21

**Authors:** Sebastian Durstberger, Klaus Widhalm, Peter Putz

**Affiliations:** FH Campus Wien – University of Applied Sciences, Department Health Sciences, Favoritenstrasse 226, 1100, Vienna, Austria

**Keywords:** Smartphone walking, 3D gait analysis, Split-belt treadmill, Instrumented treadmill, Knee abduction moment

## Abstract

**Introduction:**

In addition to its effects on cognitive awareness, smartphone use while walking may reduce the speed, regularity, and symmetry of walking. Although its effects on spatiotemporal gait parameters, such as walking speed and step width, have already been studied, little is currently known about the impact of smartphone dual tasking on lower limb kinetics.

**Research question:**

Does smartphone use during walking alter gait patterns (i.e., walking speed and step width) and consequently external knee moments?

**Methods:**

In a four-period crossover trial, external knee moment peaks, walking speed, and step width were assessed in 27 healthy adults during matched-speed walking, self-paced walking, self-paced walking with spoken calculation tasks, and self-paced walking with smartphone-entered calculation tasks. Differences between the smartphone use condition and all other conditions were determined using repeated measures ANOVA with predefined contrasts.

**Results:**

Decreased walking speed and increased step width were observed during smartphone use. The mean *external knee abduction moment peak* (Nm/kg) differed significantly (p < 0.01) across the intervention condition, namely walking with smartphone-entered calculations (0.15; 95 % CI: 0.12, 0.18), and the control conditions, namely matched-speed walking (0.11; 95 % CI: 0.08, 0.13), self-paced walking (0.11; 95 % CI: 0.09, 0.14), and walking with spoken calculations (0.14; 95 % CI: 0.12, 0.16). After confounder adjustment for walking speed, step width, gender, and age, this primary outcome was significantly different between using the smartphone and self-paced walking (p < 0.01, r = 0.51). This effect size was reduced when comparing smartphone use with spoken calculations (p = 0.04, r = 0.32).

**Conclusion:**

When using a smartphone while walking, walking speed is slowed down, step width is increased, and knee moments are adversely altered compared to walking without dual tasking. These altered knee moments are partially, but not entirely, attributable to the cognitive calculation task. These effects are age-independent, but women are more affected than men. Nevertheless, it remains unclear whether sustained walking while using a smartphone adversely affects the development of knee joint pathologies.

## Highlights

• Smartphone use while walking increases step width and thus knee abduction moment.
• The cognitive component contributes more to this effect than manual operation.
• Slower walking speeds under this condition do not fully mitigate adverse effects.

## 1 Introduction

Smartphone use while walking impairs the operator's perception of the environment [1]. This provokes inattention and thus increases the risk of accidents [2]. Apart from the altered cognitive perception [1,3], smartphone use may decrease walking speed, regularity, and symmetry [[4], [5], [6], [7], [8]]. Shorter stride lengths, longer stride times, slower gait speeds, extended stance phases, and wider step widths have been observed when using a smartphone [9,10]. However, no study has yet definitively determined which dual task involving smartphone use influences gait patterns the most. Bovonsunthonchai et al. investigated the impact of different smartphone tasks on the gait of healthy young adults. They found that tasks with a visual component affected gait more than other tasks, such as making a phone call [9]. Another study concluded that experienced smartphone users were impaired more by bimanual device holding and a limited field of view than by cognitive tasks [11]. In contrast, Shahidian et al. found that talking on the phone increased the risk of falling more than reading or writing on a smartphone [12]. In a systematic review and meta-analysis summarizing the effects of different activities on gait speed, step length, and step width, Zhang et al. [13] suggested that both writing and reading were associated with reduced gait speed and step length, whereas talking and dialing were not. However, all these activities resulted in an increase in the step width. Furthermore, Lim et al. showed that typing on a smartphone strongly reduced pedestrians' attention to their surroundings [14]. To our knowledge, no study has yet investigated the impact of walking while using a smartphone on lower limb kinetics.

Existing literature has detailed the effects of altered gait patterns on kinetics. Indeed, previous studies have shown that increasing the step width triggers an increase in the external knee abduction moment (EKAbdM) [15] but decreases the first and second external knee adduction peaks (EKAddM) [[15], [16], [17]]. Furthermore, another study showed that the walking speed was positively associated with EKAddM [18]. Van der Noort et al. observed that the increase in EKAddM was only significant at higher walking speeds, with no significant reductions at lower walking speeds [19].

However, despite existing knowledge on the effects of gait alterations on knee moments, no study has yet investigated the effects of walking while using a smartphone on knee moments. Therefore, this study aimed to investigate whether smartphone use alters EKAbdM and to what extent such changes are independent of walking speed and simultaneous cognitive tasks during smartphone use. The research question was framed to understand the impact of smartphone use on EKAbdM, hypothesizing that it would increase owing to the increased step width associated with smartphone use.

## 2 Materials and methods

### 2.1 Study design and participants

This study was set up as a hypothesis-driven four-period crossover trial that followed a randomized sequence to avoid carryover effects. A previous study had shown that walking speed affects the EKAddM peak by up to 10 % [18]. Assuming, based on the results of this study, an effect size estimate f(U) of 0.4, power of 0.8, alpha of 0.05, one group, and four conditions being studied, the sample size calculation using G*Power 3.1.9 found that a total of 25 participants were required. The following inclusion criteria were defined: (1) age 18–35 years; (2) daily smartphone use; (3) body mass index of 18.5–29.99 kg/m^2^; (4) no chronic joint disease; ligament reconstructions, or conversion osteotomy; and (5) no neuromotor disease. Recruitment was conducted at the *FH Campus Wien University of Applied Sciences*.

The ethics committee of the Medical University of Vienna approved this study (2305/2019). All participants provided written informed consent. The randomization procedure was reported in accordance with the Consolidated Standards of Research Trials (CONSORT) guidelines [20].

### 2.2 Calculation task

In this study, participants had to solve the serial seven test [21] under two different execution modes. The experimenter assigned a random starting number between 950 and 1050. They had to count backward from this starting number in steps of seven while walking. The first mode required them to say the results aloud, whereas the second execution mode required them to type the results in a smartphone. Each number they articulated or typed in the smartphone was counted as a calculation step. If the results were wrong, they continued calculating from the wrong number. Consequential errors were not counted as further errors. The error rate was determined by calculating the ratio of incorrect results to the total number of calculation steps. The subjects’ subtraction calculation performance was evaluated based on the calculation steps per minute (n/min) and the error rate (%).

### 2.3 Gait analysis

All measurements took place at the gait and movement laboratory of the *FH Campus Wien University of Applied Sciences* between March and June 2020. Initially, the participants’ height was measured to the nearest 0.5 cm using a stadiometer (SECA 213), whereas weight was measured using a medical scale (Marsden M − 420). Thereafter, all participants were equipped with 67 retroreflective markers. The extended *Cleveland Clinic Marker Set* [22] and the *Human Body Model* [23] were combined and used to control the *Gait Real-time Analysis Interactive Lab* (Motekforce Link, Amsterdam, Netherlands) [24].

All participants wore the same shoe model. The investigator explained the treadmill's self-paced mode to the participants. This mode automatically adjusts the belt speed to the participant's position on the treadmill. Before each measurement, a 4-min familiarization period was completed at a self-selected speed. The following four tasks were then performed in the order given by the randomized sequence list. Each task was split into 2-min familiarization and 3-min measurement periods. Within these measurement periods, walking was recorded three times for 10 seconds each.

• **Matched-speed walking** (condition A): Participants were instructed to walk on the treadmill without additional tasks. The walking speed was set to match that of condition D.
• **Self-paced walking** (condition B): Participants were instructed to walk on the treadmill at a self-selected speed.
• **Self-paced walking with spoken calculations** (condition C): Participants were instructed to walk on the treadmill at a self-selected speed, during which they had to continuously solve the calculation tasks and articulate the results aloud.
• **Self-paced walking with smartphone-entered calculations** (condition D): Participants were instructed to walk on the treadmill at a self-selected speed, during which they had to continuously solve the calculation tasks, hold the smartphone with both hands, and enter the calculation results manually.

All marker trajectories were filtered using a *Woltring* lowpass filter (mean square error = 20) [25]. Force plate data were smoothed using a *Butterworth* fourth order lowpass filter with a cutoff frequency of 12 Hz. No phase shift was caused by the filtering. Gait events were detected at a threshold of 20 N from the vertical ground reaction force. The gait cycle was time-normalized to 100 % between two initial contacts of the same foot. Six gait cycles from each condition were averaged and used for analysis. The hip joint center was estimated according to the regression equation proposed by Davis [26]. The knee joint center and ankle joint center were determined to be the midpoints of the lateral and medial markers on the femur condyles and malleoli, respectively [27]. An inverse dynamic approach was used to determine external joint moments with the Newton–Euler formula [26]. The reference system for joint moments was the orthogonal coordinate system of the distal joint segment [26]. The guidelines of the International Society of Biomechanics [24] for the calculation and reporting of the external frontal knee moment were carefully considered. The primary outcome parameter was the EKAbdM peak normalized to body mass (Nm/kg). This parameter was determined as the most prominent external abduction value during the stance phase [28]. The first EKAddM peak (maximal adduction value during the first half of the stance phase), second EKAddM peak (maximal adduction value during the second half of the stance phase), external knee flexion moment peak (EKFM; maximal flexion value during the first half of the stance phase), external knee extension moment peak (EKEM; maximal extension value during the second half of the stance phase), walking speed (m/s), and step width (m) were additionally assessed as secondary outcomes.

### 2.4 Randomization procedure

Three of the four conditions (conditions B, C, and D) were tested under block randomization of six possible permutations (BCD, BDC, CBD, CDB, DBC, and DCB). The fourth corresponding condition (walking at a speed matching that of condition D) could technically not occur before the corresponding smartphone condition and was, therefore, exempted from the sequence randomization and always followed self-paced walking with smartphone-entered calculations.

### 2.5 Statistical methods

All parameters were analyzed with SPSS 27.0 (IBM Corporation, Armonk, NY, USA), and the effect size (ω^2^) was calculated using Microsoft Excel 2016 Professional. One-factorial repeated measures analysis of variance (rmANOVA) was used to assess differences in the EKAbdM and secondary outcomes between the four conditions. Given that participants presented without affected legs, one side per subject was randomly selected for evaluating the outcomes of knee kinetics. The normality assumption, homogeneity of variances, and sphericity were determined using the Shapiro–Wilk test, Levene test, and Mauchly test, respectively. Subtraction calculation performances in conditions B and C were tested for normality using the Shapiro–Wilk test, whereas differences between these conditions were determined using the paired *t*-test or Wilcoxon test. Moment waveforms were compared using rmANOVA based on a one-dimensional statistical parameter mapping model (spm1d) [29].

The following overall primary null hypothesis was tested using rmANOVA:

The means of the outcome EKAbdM peak would be equal across the four conditions. Our predefined contrast null hypotheses were as follows: (1) the outcome of condition A would be equal to that of condition D; (2) the outcome of condition B would be equal to that of condition D; and (3) the outcome of condition C would be equal to that of condition D. The alternative hypotheses assumed inequality in each case.

For the overall rmANOVA, the effect size (ω^2^) was calculated from the test statistic F and interpreted as small, medium, and large when ω^2^ was ≥0.01, ≥0.06, and ≥0.16, respectively [30]. Contrast effect sizes (r) were calculated as point–biserial correlations from the test statistics F and interpreted as small, medium, and large when r was ≥0.1, ≥0.3, and ≥0.5, respectively [31]. The paired *t*-test effect size (Cohen's d) was interpreted as small, medium, and large when d was ≥0.2, ≥0.5, and ≥0.8, respectively [31].

An additional exploratory analysis was performed to obtain contrast p-values and standardized effect sizes (r), with a confounder adjustment for walking speed, step width, gender, and age. Due to time-varying covariates (walking speed and age), this was facilitated using the SPSS MIXED linear procedure, with the data arranged in the long format, i.e., one row per time point per subject, with a subject identification variable. Huynh-Feldt was specified as the covariance type, and standardized effect sizes (r) were calculated as SQRT (F/(F + df), where “F” is the test statistic and “df” is Satterthwaite's approximate degrees of freedom. In addition to the condition solely as factor (equal to the contrasts without adjustment for confounders), four separate models were run with the interactions as follows: 1) condition*walking speed, 2) condition*walking speed*step width, 3) condition*walking speed*step width*gender, and 4) condition*walking speed*step width*gender*age.

Alpha was set to 0.05 and corrected using the Bonferroni–Holm method. Thus, in a particular case, the smallest contrast p value was multiplied by three and the second smallest p value by two, thereby correcting for the multiplicity of testing within, for instance, the primary hypothesis model. Similarly, the secondary outcomes were corrected for multiplicity of testing.

## 3 Results

### 3.1 Characteristics of study participants

A total of 29 healthy subjects (18 women and 11 men) were recruited to participate in this study. However, after excluding two women from data processing, 27 subjects (mean age, 25.3 years) were ultimately included in the analysis. Given that the usual gait pattern of the excluded women had a minimal step width, it was impossible to record a sufficient number of correct force plate strikes on the treadmill (Table 1).

**Table 1:** Age and anthropometric characteristics, mean (SD).

	n	Age (years)	Weight (kg)	Height (cm)	**BMI**^a^**(kg m**^**−2**^**)**
*Women*	16	23.6 (4.3)	64.8 (6.3)	168.7 (5.2)	22.7 (1.4)
*Men*	11	27.6 (5.2)	72.4 (9.7)	176.8 (8.8)	23.1 (1.9)
*All*	27	25.3 (5.0)	67.9 (8.6)	172.0 (7.8)	22.9 (1.6)

a Body mass index based on measured data.

### 3.2 Calculation task

The boxplot in Fig. 1 shows that the participants managed more calculations per minute with mental calculations [mean (M) = 15.40, standard error (SE) = 0.90] than with smartphone-entered calculations (M = 13.16, SE = 1.04). The mean difference was 2.24 (95 % CI: 0.01, 4.38), which was determined to be significant [T (26) = 2.148, p = 0.041] and represented a small effect size of d = 0.413. However, no significant differences in error rates were observed between mental and smartphone-entered calculations.

**Fig. 1:**
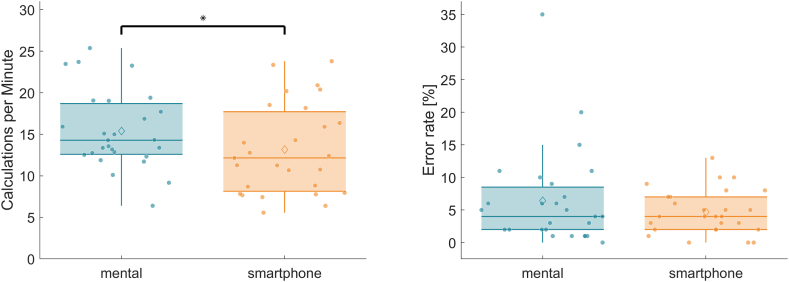
Boxplots (n = 27) for mental calculation and smartphone calculation for the parameter calculations per minute (left) and error rate (right). * indicates significant difference (paired t-test, p < 0.05).

### 3.3 3D gait analysis

**Fig. 2:**
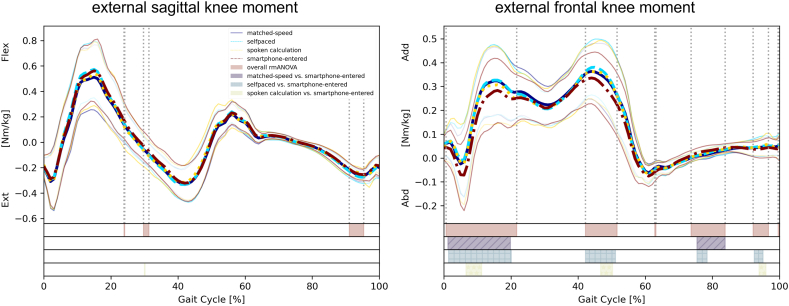
Averaged external sagittal and frontal knee moment for all four conditions, with standard deviation. Bars at the bottom of the plots show spm1d results, regarding the overall rmANOVA and predefined contrasts.

The Shapiro–Wilk test rejected the assumption that the primary outcome had a normal distribution. Nevertheless, rmANOVA was applied given its robustness to the violation of the normality assumption, as described by Khan and Rayner [32]. Additional ln-transformed model calculations did not affect the statistical significance (data not shown). Mauchly's test confirmed the assumption of sphericity, except for the EKAbdM peak and the second EKAddM peak. Consequently, these two parameters were calculated using Greenhouse–Geisser's corrected degrees of freedom. Fig. 2 shows the averaged waveforms of the sagittal and frontal knee moments, with the bars at the bottom of the plots showing the spm1d results. The averaged waveforms of the hip moment are provided in the Appendix. The EKAbdM peak and the step width were highest in condition D, wherein smartphone-entered calculations were performed at a self-paced walking speed. In condition B (i.e., self-paced walking), the participants had the highest walking speed.

### 3.4 Primary outcome ANOVA results and contrasts

The overall primary rmANOVA (Table 2) showed that the mean *EKAbdM peak* differed across the studied conditions [F (3, 26) = 15.24, p < 0.001], which represented a medium-sized effect (ω^2^ = 0.059). Analysis of predefined contrasts in Table 3 showed that smartphone-entered calculations caused a significantly greater increase in EKAbdM than did matched-speed walking (contrast 1: adjusted p < 0.001, r = 0.856). Performing calculations at a self-selected walking speed had a marginally smaller effect (contrast 2: adjusted p < 0.001, r = 0.764). However, no significant difference in the primary outcome was observed between smartphone-entered and spoken calculations (contrast 3: adjusted p = 0.269, r = 0.216).

**Table 2:** Knee abduction moment peak and secondary outcomes across the studied conditions.

		Condition A mean (95 % CI)	Condition B mean (95 % CI)	Condition C mean (95 % CI)	Condition D mean (95 % CI)	p value	Adjusted p value	ω^2^
*Primary outcome*	*External knee abduction moment peak (Nm/kg)*	0.106 (0.082, 0.129)	0.113 (0.088, 0.138)	0.136 (0.116, 0.156)	0.147 (0.119, 0.175)	<0.001		0.059
*Secondary outcomes*	*First external knee adduction moment peak (Nm/kg)*	0.382 (0.335, 0.428)	0.386 (0.337, 0.434)	0.383 (0.330, 0.437)	0.346 (0.300, 0.391)	<0.001	<0.001	0.016
	*Second external knee adduction moment peak (Nm/kg)*	0.401 (0.350, 0.452)	0.412 (0.363, 0.462)	0.408 (0.354, 0.461)	0.369 (0.324, 0.413)	<0.001	<0.001	0.015
	*External knee flexion moment peak (Nm/kg)*	0.583 (0.480, 0.686)	0.650 (0.567, 0.733)	0.634 (0.538, 0.731)	0.630 (0.532, 0.727)	0.023	0.460	0.007
	*External knee extension moment peak (Nm/kg)*	0.370 (0.322, 0.419)	0.374 (0.323, 0.426)	0.371 (0.318, 0.424)	0.362 (0.307, 0.417)	0.785	0.785	0.000
	*Walking speed(m s*^*−1*^*)*	1.37 (1.28, 1.45)	1.48 (1.41, 1.55)	1.41 (1.35, 1.47)	1.36 (1.24, 1.45)	0.001	0.001	0.046
	*Step width (m)*	0.070 (0.063, 0.078)	0.077 (0.070, 0.084)	0.082 (0.072, 0.092)	0.092 (0.082, 0.102)	<0.001	<0.001	0.101

p values were derived from repeated measures ANOVA. Condition A: matched-speed walking; Condition B: self-paced walking; Condition C: self-paced walking with spoken calculation: Condition D: self-paced walking with smartphone-entered calculation; 95 % CI: 95 % confidence interval; ω^2^: effect size omega^2^ from repeated measures ANOVA.

^a^Greenhouse–Geisser adjusted.

**Table 3:** External knee abduction moment peak contrast and contrast of secondary outcomes determined to be significant on repeated measures ANOVA.

	Contrast	Unadjusted p value	Bonferroni–Holm adjusted p value	r	Interpretation
***External knee abduction moment peak***	A vs. D (contrast 1)	<0.001	**<0.001**	0.856	large
	B vs. D (contrast 2)	<0.001	**<0.001**	0.764	large
	C vs. D (contrast 3)	0.269	0.269	0.216	small
***External knee adduction moment first peak***	A vs. D (contrast 1)	<0.001	**<0.001**	0.685	large
	B vs. D (contrast 2)	<0.001	**<0.001**	0.742	large
	C vs. D (contrast 3)	<0.001	**<0.001**	0.613	large
***External knee adduction moment second peak***	A vs. D (contrast 1)	0.014	**0.014**	0.457	medium
	B vs. D (contrast 2)	<0.001	**<0.001**	0.690	large
	C vs. D (contrast 3)	<0.001	**0.001**	0.602	large
***External knee flexion moment peak***	A vs. D (contrast 1)	0.004	**0.011**	0.532	large
	B vs. D (contrast 2)	0.351	0.703	0.183	small
	C vs. D (contrast 3)	0.836	0.836	0.041	negligible
***Walking speed***	A vs. D (contrast 1)	0.261	0.261	0.220	small
	B vs. D (contrast 2)	0.001	**0.004**	0.577	large
	C vs. D (contrast 3)	0.189	0.377	0.256	small
**Step width**	A vs. D (contrast 1)	<0.001	**<0.001**	0.700	large
	B vs. D (contrast 2)	0.003	**0.006**	0.544	large
	C vs. D (contrast 3)	0.083	0.083	0.333	medium

Condition A: matched-speed walking; Condition B: self-paced walking; Condition C: self-paced walking with spoken calculation; Condition D: self-paced walking with smartphone-entered calculation.

### 3.5 Secondary outcome ANOVA results and contrasts

As shown in Table 2, the mean *first EKAddM peak* differed between the groups [F (3, 26) = 11.04, adjusted p < 0.001], with a small-sized effect (ω^2^ = 0.016). Analysis of contrasts revealed significant differences for all three contrasts (contrast 1: adjusted p < 0.001, r = 0.685; contrast 2: adjusted p < 0.001, r = 0.742; contrast 3: adjusted p < 0.001, r = 0.613).

Similar to the first peak, the mean *second EKAddM peak* differed between the groups [F (3, 26) = 7.54, adjusted p < 0.001], with a similar small-sized effect (ω^2^ = 0.015). Analysis of contrasts revealed significant differences for contrast 1 (adjusted p = 0.014, r = 0.457), contrast 2 (adjusted p = 0.003, r = 0.570), and contrast 3 (adjusted p = 0.002, r = 0.602).

Furthermore, differences in *walking speed* [F (3,26) = 5.369, adjusted p < 0.010] with a small-sized effect (ω^2^ = 0.046), *step width* [F (3, 26) = 10.057, adjusted p < 0.001] with a medium-sized effect (ω^2^ = 0.101), and *EKFM peak* [F (3, 26) = 3.34, adjusted p = 0.046] with a negligible effect size (ω^2^ = 0.007) were observed between the groups. *EKEM peak* [F (3, 26) = 0.25, adjusted p = 0.78, ω^2^ = −0.002] was equal across the studied conditions.

### 3.6 Confounder adjusted contrast analyses

Table 4 provides an overview of whether and to what extent contrast effects (Table 3) were confounded by walking speed, step width, gender, and age. For the primary outcome *EKAbdM peak, e*ffect size interpretations and corresponding significance remained unchanged for contrast 1 and contrast 2, although the effect sizes were somewhat reduced by the correction for walking speed and gender. Contrast 3 became statistically significant, and the effect sizes increased from small to moderate by the correction for walking speed and step width. Adjusting for age showed no relevant confounding.

**Table 4:** External knee abduction and secondary outcomes confounder adjusted contrasts.

		Model without confounder adjustment		Model condition*walking speed		Model condition*walking speed*step width		Model condition*walking speed*step width*gender		Model condition*walking speed*step width*gender*age	
	Contrast	**p**	**r**	p	r	p	r	p	r	**p**	**r**
***Ext. knee abduction moment peak***	A vs. D	**<0.001**	**0.86**	<0.001	0.73	<0.001	0.77	<0.001	0.53	**<0.001**	**0.55**
	B vs. D	**<0.001**	**0.76**	<0.001	0.68	<0.001	0.67	<0.001	0.49	**<0.001**	**0.51**
	C vs. D	**0.269**	**0.22**	0.060	0.29	0.022	0.35	0.070	0.29	**0.039**	**0.32**
***Ext. knee adduction moment* 1st *peak***	A vs. D	**<0.001**	**0.69**	<0.001	0.50	<0.001	0.50	0.088^**BH**^	0.30	**0.146**^**BH**^	**0.28**
	B vs. D	**<0.001**	**0.74**	<0.001	0.61	<0.001	0.63	0.012^**BH**^	0.40	**0.012**^**BH**^	**0.40**
	C vs. D	**<0.001**	**0.61**	0.002	0.48	0.003	0.45	0.273	0.19	**0.400**	**0.16**
***Ext. knee adduction moment 2nd peak***	A vs. D	**0.014**	**0.46**	0.032	0.34	0.017	0.36	0.006^**BH**^	0.41	**0.008**^**BH**^	**0.39**
	B vs. D	**<0.001**	**0.69**	<0.001	0.54	<0.001	0.58	<0.001	0.46	**<0.001**	**0.45**
	C vs. D	**<0.001**	**0.60**	<0.001	0.50	<0.001	0.49	0.280	0.06	**0.090**	**0.26**
***Ext. knee flexion moment peak***	A vs. D	**0.012**^**BH**^	**0.53**	<0.001	0.50	0.003^**BH**^	0.50	0.003^**BH**^	0.44	**0.006**^**BH**^	**0.42**
	B vs. D	**0.702**^**BH**^	**0.18**	<0.001	0.49	0.084	0.29	0.034^**BH**^	0.35	**0.058**^**BH**^	**0.33**
	C vs. D	**0.836**	**0.04**	0.002	0.45	0.010^**BH**^	0.33	0.154	0.24	**0.216**	**0.22**

Condition A: matched-speed walking; Condition B: self-paced walking; Condition C: self-paced walking with spoken calculation; Condition D: self-paced walking with smartphone-entered calculation.

^BH^ … Bonferroni-Holm adjusted p-values (values not marked with ^BH^ remained unchanged after the Bonferroni-Holm adjustment).

Bold values highlight the results of the model without adjustment for confounders and the fully adjusted model (all confounders included).

Similarly, *EKFM peak* interpretations remained unchanged or even increased in effect size after adjustment for confounders.

However, this confounder adjustment revealed reduced effect sizes in all contrasts for the *EKAddM* *peaks*, due to the correction for walking speed and gender, whereas no relevant confounding was identified by step width and age.

## 4 Discussion

This study investigated how using a smartphone affects knee moments, a topic that has not been explored previously. To assess the impact of gait speed on the study outcomes, two control conditions were employed, one with matched speed (condition A) and the other with a self-selected speed (condition B). A third control condition (condition C) was included to evaluate the effects of the cognitive tasks. The findings obtained in this study in terms of altered gait speed and step width when walking while using a smartphone are consistent with those reported in previous research [[4], [5], [6], [7], [8]]. In fact, our comparisons between smartphone-based calculations and adjusted speed as well as smartphone-based calculations and self-selected speed revealed significant differences, which demonstrated large effect sizes. This finding suggests that gait speed does not affect the primary outcome. These observations agree with those reported in the study by van der Noort et al. [19], which showed that higher gait speeds make a difference, whereas lower gait speeds do not. Although walking while using a smartphone reduces the gait speed, the *EKAbdM peak* observed in condition D was higher than that observed while walking at matched and self-selected speeds. The comparison between spoken and smartphone calculations revealed no significant difference in the *EKAbdM peak*, in the experimental setting. Thus, the simultaneous cognitive task might have exerted a stronger influence on knee moments compared with operating the smartphone itself. In contrast, Marone et al. [11] concluded that experienced texters might be affected by physical but not cognitive demands of texting under controlled treadmill walking conditions. However, the aforementioned study examined the frontal margin of stability and not *EKAbdM*.

An analysis of the step width results revealed significant differences between walking while using a smartphone (condition D) and walking under matched speed (condition A) and self-selected speed (condition B) conditions. Although walking while engaging in a cognitive task (condition C) resulted in a moderate effect (r = 0.333) on stride width, the increase was not significant. These findings allude that cognitive tasks had a slight impact on stride width, whereas walking while using a smartphone had a more noticeable effect, leading to greater deviations in the stride width.

We observed a reduction in the *EKAddM peaks* when walking while using smartphones. The first peak was reduced during the smartphone-entered calculations but was similar across the other three conditions. Therefore, we can conclude that walking while using a smartphone reduces the first external *EKAddM* independent of the cognitive task and gait speed. Likewise, the second *EKAddM peak* was lowest when walking while performing smartphone-entered calculations. However, the confounder adjusted models showed that gender was a strong confounder. The observed effects were stronger in female participants. Due to the unbalanced distribution in our sample, the effect was therefore overestimated. Thus, at the first peak, the adjusted model showed no significant difference to the matched speed condition. Compared to the spoken-calculation condition, the adjusted model showed no significant difference for the first and second peak. Furthermore, this analysis showed that walking speed played a role as a confounder in comparison to the speed-matched condition. Because the speed matching was set based on an average speed throughout the trial, the speed could vary slightly during the measurement periods. A significant difference in the *EKFM peak* was observed only when comparing walking while performing calculations and walking at a matched speed.

The results of the spm1d analysis generally agreed with those of the discrete parameter analysis. However, some differences between the two methods were observed in the post-hoc analysis. For instance, the post-hoc test of the spm1d analysis did not reveal any significant differences between self-paced walking and walking while performing smartphone-entered calculations at the time of the second *EKAddM peak*. In contrast, discrete value analysis did reveal a significant difference with a medium effect size. It is worth noting, however, that the timing at which the peak occurred had no influence on discrete value analysis but did play a critical role in the spm1d analysis.

The step width may increase the *EKAbdM peak* and decrease the *EKAddM peak* only marginally. In line with this finding, Stief et al. showed that an increase in the step width reduces *EKAddM* [15]. The literature describes trunk lean as a compensatory movement made by patients with knee osteoarthritis to alter their external frontal knee moments [33,29]. Although outside of the scope of this study, we assume that the absence of an arm pendulum when holding a smartphone bimanually causes a change in the upper body that also affects the external frontal knee moments.

Lamberg and Mauratori [34] described a decrease in walking speed when texting on a smartphone, which they attributed to the cognitive distraction of a dual task. Lee and Jeon [35] described the effect of walking while using a smartphone on the muscle activity of the lower extremities. They also found a reduced walking speed and identified a potential risk for musculoskeletal problems. Electromyography showed a decrease in the muscle activity of the gluteus maximus and medius, biceps femoris, rectus femoris, gastrocnemius, and tibialis anterior. Based on these results, they concluded that frequent smartphone use while walking can be a risk factor for musculoskeletal disorders. Although our results show a change in kinetics from normal walking, we could not definitively determine whether the change in external frontal knee moments has clinically relevant consequences. Likewise, we could not determine whether sustained walking while using a smartphone has adverse or favorable effects on the development of knee joint pathologies. Compared with walking without a smartphone, walking while using the device increases the *EKAbdM* at the beginning of the stance phase and decreases it during the rest of the stance phase. Further research using musculoskeletal modeling could investigate how this pattern affects the load on the medial and lateral knee contact area.

A major strength of the study was the design, which included three control conditions, which made it possible to assess the extent to which the effects were caused by the manual operation of the smartphone itself and the extent to which they were confounded by the simultaneous reduction in walking speed and cognitive calculation tasks.

One limitation of this study is that performing mental calculations on a smartphone is not a common everyday task. Therefore, our findings may not be directly transferable to everyday tasks such as writing an email, making a video call, or playing a game. Caramia et al. [4] showed in their study that solving mathematical tasks on a smartphone leads to high dual-task costs, which are comparable to surfing the Internet and higher than writing a short text such as a message. In addition, the participants did not use their personal smartphones but were instead provided devices of an identical model. Therefore, some subjects might not have used their usual hand position. Participation in the study was open to all students and staff of the FH Campus Wien University of Applied Sciences. However, most of the participants were physiotherapy students. Given this background, most subjects were trained in posture and movements. The sample was rather small (n = 27) and not fully gender balanced. Hence, the generalizability of the findings may be limited. The assessments were performed on an instrumented treadmill, and care was taken to ensure that each subject received an adequate familiarization time of 6 min [36]. However, some individuals might have altered their natural gait patterns owing to the treadmill setting. Van der Krogt et al. showed that treadmill kinetics differ from ground walking kinetics [37]. Therefore, caution should also be exercised when generalizing these laboratory findings to ground walking.

Future research would ideally use prospective observational designs that track the development of knee joint pathologies and include a forward model of contact forces. Experimental prospective designs could examine the effectiveness of interventions such as reduced smartphone use while walking or specific gait training interventions for smartphone use. To better understand the mechanisms behind altered knee moments caused by smartphone use while walking, studies should account for the level of consciousness, cognitive state, and alertness in different real-life smartphone use scenarios, such as making video calls or playing games.

## 5 Conclusion

When using a smartphone while walking, walking speed is slowed down, step width is increased, and the *EKAbdM peak* is largely increased while the *EKAddM peak* is moderately decreased compared to walking without dual tasking. These effects are partially, but not entirely, attributable to the cognitive calculation task. They are age-independent, but women are more affected than men. Whether adverse or favorable effects on the development of knee joint pathologies are exerted by sustained walking while using a smartphone remains to be elucidated.

## Ethics statement

The ethics committee of the Medical University of Vienna approved this study (2305/2019). All participants provided written informed consent.

## Funding sources

This research did not receive any specific grant from funding agencies in the public, commercial, or not-for-profit sectors.

## Data availability statement

Data will be made available on request.

## CRediT authorship contribution statement

**Sebastian Durstberger:** Writing – original draft, Software, Project administration, Methodology, Investigation, Formal analysis, Data curation. **Klaus Widhalm:** Writing – review & editing, Software, Methodology, Investigation, Formal analysis, Data curation, Conceptualization. **Peter Putz:** Writing – review & editing, Supervision, Methodology, Investigation, Formal analysis, Data curation, Conceptualization.

## Declaration of competing interest

The authors declare that they have no known competing financial interests or personal relationships that could have appeared to influence the work reported in this paper.

## Figures and Tables

**figs1:**
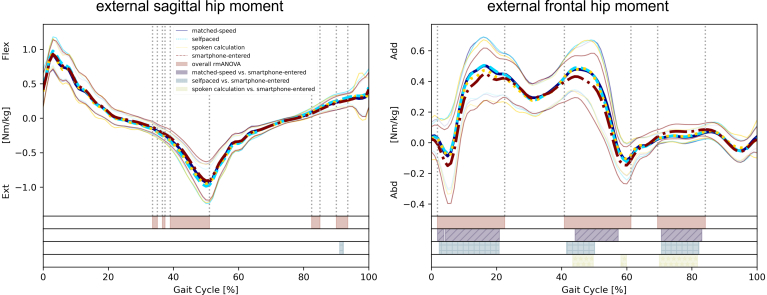
Averaged external sagittal and frontal hip moment for all four conditions, with standard deviation.Bars at the bottom of the plots show spm1d results, regarding the overall rmANOVA and predefined contrasts.
